# Trends and Prospects in Pig Genomics and Genetics

**DOI:** 10.3390/genes15101292

**Published:** 2024-09-30

**Authors:** Katarzyna Piórkowska, Katarzyna Ropka-Molik

**Affiliations:** National Research Institute of Animal Production, Animal Molecular Biology, 31-047 Cracow, Poland; katarzyna.ropka@iz.edu.pl

Pork is one of the most commonly consumed meat in the world. The domestication of pigs in Europe dates back to 5000 BC, from which the selection over decades has led to a significant reduction in backfat levels and improvements in growth rates. In pigs, with the development of registered and genetic evaluation methods, breeding has moved from genetic improvement through open herd books to breeding-specific lines [1]. Breeding goals in Europe have changed for economic reasons, consumer tastes and customs, and also due to the introduction of new methods for measuring phenotypic traits. Today’s goals focus on reproduction, resistance, and meat quality. Nevertheless, work is still underway on new breeding goals based on important new traits, as well as their study and implementation in measurement tools [2]. Genomic selection used in many countries can increased the genetic progress rate by up to 35% per year for all traits. However, not all European countries have introduced this requirement; in many, activities are still underway, e.g., preparing reference populations (Relationship-Based Genomic Selection (RBGS)) [3]. Proposed the next step in pig breeding is to use full information on the DNA sequence, which will enable an understanding of specific genotypes and their underlying biology, as well as identifying genes that may affect resistance to diseases that pose a challenge to the industry [4]. Another advance in the genetic improvement of pigs was the attempt at gene editing, where based on these methods, pigs resistant to PRRSv were produced [5]. Geneticists’ current challenge is developing pigs resistant to ASF, a disease brought from Africa that causes huge economic losses, mainly among the populations of central and eastern Europe [6,7].

There is still a lot going on in pig genetics and genomics, not only in Europe. The latest research on Chinese pig populations, based on the genome at the chromosomal level of the Chenghua pig, has redefined pig introgression [8]. It has been determined that the migration routes of pigs from China, where pig domestication occurred much earlier than in Europe, approximately 12,000 years ago, led gradually through different geographical areas of China and only then reached Europe. In addition, the researchers identified two genes, FBN1 and NR6A1, associated with evolutionary adaptation to different geographical latitudes in Chinese pigs [8]. Another Chinese study based on 250 sequences from 32 Eurasian pig breeds constructed a pangenome in which the so-called PAVs (presence/absence variants), non-reference sequences, and over 3000 new genes were identified, as well as unidentified features of the pig mobilome, including several transposable elements (TE) candidates as adaptive insertions that were co-opted into genes responsible for hypoxia responses, skeletal development, regulation of heart contraction and development of neurons, probably contributing to the local adaptation of Tibetan wild boars [9].

This special issue, entitled Trends and Prospects in Pig Genomics and Genetics, examines various directions for improving this species in terms of utility, health, and welfare traits, as well as the use of porcine cells to model human disease states.

One of the main directions of the current SI was the assessment of molecular mechanisms related to the deposition of adipose tissue, which is associated with the taste of pork, but is also considered in the context of modelling processes generally related to obesity.

Blińska et al. [10] used an in vitro model of differentiation of mesenchymal stem cells into adipocytes and digital PCR to identify significant extra- and intracellular microRNA potential involved in adipogenesis in their study. They observed that only microRNAs related to the inflammatory process were highly expressed and secreted by differentiating adipocytes. On the other hand, Wang et al. [11] focused on LncPLAAT3-AS transcript, which promotes the transcription of PLAAT3, a regulator of adipogenesis. Using dual luciferase assay, the authors revealed that LncPLAAT3-AS sponging miR-503-5p leads to the inhibition of PLAAT3 expression. Moreover, additional observation showed that LncPLAAT3-AS and PLAAT3 were significantly more expressed in fatty pig breeds’ adipose tissue than in lean pigs. In turn, using RT-PCR and RACE methods, Zhu et al. [12] characterized in fat tissue cis-SAGe product BCL2L2–PABPN1 (PB), generated based on the fusion of both proteins. It was found that PB promotes proliferation and inhibits the differentiation of primary porcine preadipocytes. Moreover, in porcine preadipocytes with overexpression of PB, identified numerous DEGs (differentially expressed genes) and DEmiRNAs related to fat-associated pathways such as MAPK and PI3K-AKt and miRNAs ssc-miR-339-3p is critical for adipogenesis regulation throughout PB. Lin et al. [13] recognized the role of SESN3 in regulating adipogenesis and found that Sestrin-3 inhibits porcine preadipocyte proliferation. The mechanism of activation was through SMAD3 involved in the development of non-alcoholic steatohepatitis (NASH), where SESN3 inhibited SMAD3, thus improving ssc-miR124 activity, and then suppressed C/EBPα and GR to regulate pre-adipocytes adipogenesis. Piórkowska et al. [14] using variant calling and χ2 analyses based on liver RNA-seq data, identified genetic markers related to the FGL1 gene, which probably play a significant role in lipid metabolism because it is related to the regeneration of the liver organ, in addition, its abundance is expressed in brown adipose tissue and associated with proper plasma lipid profiles in mice blood. The authors suggested that the FGL1 rs340465447_A allele can be used as a target in pig selection focused on elevated fat levels.

Two reports of SI considered a problem of fertility—litter size. Srihi et al. [15] estimated the additive and dominance variances of the purebred (Retinto and Entrepelado pigs) and CASTÚA crossbreed populations for litter size (including total number born (TNB) and number born alive (NBA)) for and calculated the additive genetic correlations between the purebred and crossbred performances. The authors identified four genomic regions (containing 30 SNPs) that each explained >2% of the additive genetic variance in chromosomes 6, 8 and 12. In turn, Sell-Kubiak et al. [16] based on 2000 SNP associated with pig litter size traits and reported based on previous genome-wide association studies (GWASs), gathered and integrated associations between SNPs and these traits. Authors selected the most interesting candidate genes reported in multiple populations such as PRKD1 involved in angiogenesis and associated with stillborn and TNB, and two new not previously reported—FAM13C and AGMO related to TNB, and the most promising candidate genes for litter size—SOSTDC1, which was described before as associated with male fertile in rats.

Porcine cells are commonly used as models of human disease, which is under consideration in medical science. The present SI contains two studies [17,18] using porcine atrial cardiomyocytes during the primary in vitro culture as a model that describes molecular mechanisms occurring in these cell types related to heart failure. The heart was considered a non-regenerated organ, but a few reports suggest it has modest intrinsic regenerative potential. Therefore, Bryl et al. [18] characterized cell cultures from the right atrial appendage and right atrial wall during cell culture cultivation duration of up to 30 days, based on a microarrays approach, they observed DEG enrichment in GO of stem cell population maintenance” and “stem cell proliferation”, which suggested for previously described regenerative heart potential. In turn, Nawrocki et al. [17], using in vitro cell culture, extracted from the myocardium, revealed that DEGs were classified as involved in ontological groups such as: “cellular component assembly”, “cellular component organization”, “cellular component biogenesis”, and “cytoskeleton organization with significantly increased expression of COL5A2, COL8A1, and COL12A1 encoding different collagen subunits, pivotal in cardiac extracellular matrix (ECM) and significant down-regulated related to cellular architecture such as ABLIM1, TMOD1, XIRP1, and PHACTR1. Using porcine cells as a model allows for a better understanding of underlying molecular mechanisms of cardiovascular pathologies, which seems crucial to developing effective therapeutic options.

In this special issue, we present the latest advances in pig genetics and genomics, including identifying mechanisms related to adipose tissue deposition and lipid metabolism and using pig cells to model processes related to circulatory system abnormalities, including identifying candidate genes. The special issue also includes manuscripts on identifying markers for reproductive traits in pigs, which is an important economic aspect, and several items presenting non-standard research methods. We warmly encourage you to familiarize yourself with our new SI entitled “Trends and Prospects in Pig Genomics and Genetics”.
